# Human Chorionic Gonadotropin monotherapy for the treatment of hypogonadal symptoms in men with total testosterone > 300 ng/dL

**DOI:** 10.1590/S1677-5538.IBJU.2019.0132

**Published:** 2019-01-29

**Authors:** Vinayak Madhusoodanan, Premal Patel, Thiago Fernandes Negris Lima, Jabez Gondokusumo, Eric Lo, Nannan Thirumavalavan, Larry I. Lipshultz, Ranjith Ramasamy

**Affiliations:** 1 University of Miami Miller School of Medicine Miami FL USA University of Miami Miller School of Medicine, Miami, FL, USA;; 2 Department of Urology University of Miami Miller School of Medicine Miami FL USA Department of Urology, University of Miami Miller School of Medicine, Miami, FL, USA;; 3 Scott Department of Urology Baylor College of Medicine Houston TX USA Scott Department of Urology, Baylor College of Medicine, Houston, TX, USA

**Keywords:** Testosterone, Chorionic Gonadotropin, Hypogonadism

## Abstract

**Purpose:**

The 2018 American Urological Association guidelines on the Evaluation and Management of Testosterone Deficiency recommended that 300 ng/dL be used as the threshold for prescribing testosterone replacement therapy (TRT). However, it is not uncommon for men to present with signs and symptoms of testosterone deficiency, despite having testosterone levels greater than 300 ng/dL. There exists scant literature regarding the use of hCG monotherapy for the treatment of hypogonadism in men not interested in fertility. We sought to evaluate serum testosterone response and duration of therapy of hCG monotherapy for men with symptoms of hypogonadism, but total testosterone levels > 300 ng/dL.

**Materials and Methods:**

We performed a multi-institutional retrospective case series of men receiving hCG monotherapy for symptomatic hypogonadism. We evaluated patient age, treatment indication, hCG dosage, past medical history, physical exam findings and serum testosterone and gonadotropins before and after therapy. Descriptive analysis was performed and Mann Whitney U Test was utilized for statistical analysis.

**Results:**

Of the 20 men included in the study, treatment indications included low libido (45%), lack of energy (50%), and erectile dysfunction (45%). Mean testosterone improved by 49.9% from a baseline of 362 ng/dL (SD 158) to 519.8 ng/dL (SD 265.6), (p=0.006). Median duration of therapy was 8 months (SD 5 months). Fifty percent of patients reported symptom improvement.

**Conclusions:**

Treatment of hypogonadal symptoms with hCG for men who have a baseline testosterone level > 300 ng/dL appears to be safe and efficacious with no adverse events.

## INTRODUCTION

Hypogonadism is prevalent, currently affecting 38% of men over the age of 45, and 7% of men under the age of 40 (1, 2). Adult-Onset Hypogonadism is defined as having low testosterone in conjunction with clinical signs or symptoms such as low energy, low libido, decreased lean body mass, erectile dysfunction, fatigue, depression, anemia and infertility (3-6). Although it is primarily a clinical syndrome of the aging male (3), prevalence in younger generations has increased exponentially over the past decades, a trend that correlates with an increase in the prevalence of anabolic androgenic steroid use (1). It can be associated with various acute and chronic illnesses, and should be recognized and treated (7).

Despite the prevalence of hypogonadism, there are limited treatment options. Most often, testosterone replacement therapy (TRT) is offered to these men (3). It can be effective in improving sexual function, bone mineral density, muscle strength, energy, cognition and vitality (4, 7). However, per the recent American Urological Association (AUA) guidelines, it should be reserved for patients with testosterone deficiency, as defined as less than 300 ng/dL (8). This recommendation was determined based on a compromise between the inclusion criteria testosterone (less than 350 ng/dL) and the median testosterone levels (250 ng/dL) of most large testosterone therapy trials over the past decade, in part to minimize overtreatment of patients (8).

Unfortunately, this means that the scope of treatment with TRT can be limited, precluding treatment of patients with subclinical hypogonadism (SH), who may present with the clinical syndrome, although baseline testosterone levels remain above 300 ng/dL (9). Per the European Male Ageing Study, the prevalence of SH is close to 10%, increasing up to 21% in the 8th decade of life (9). Moreover, although TRT can be efficacious, it can be undesirable due to associated side effects and complications, including acne, gynecomastia, testicular atrophy, male infertility and increased hematocrit (4, 7).

Human chorionic gonadotropin (hCG) can be a promising alternative for these patients. This pharmacotherapy can be self-administered subcutaneously on a scheduled basis. It acts as an analogue of luteinizing hormone (LH), with the added benefit of a longer half-life (1, 4). As LH would do, hCG acts on Leydig cells, stimulating them to produce and release intratesticular testosterone (1).

Despite its promise, scant literature exists on use of hCG as a monotherapy for patients with suspicion of late-onset hypogonadism, whose primary indication for therapy is not fertility. The purpose of this study was to assess the efficacy and safety of its use in such a population. We sought to evaluate the response of serum testosterone to hCG monotherapy as evidence of its efficacy at various doses and therapeutic durations, as well as its safety. We hypothesize that hCG monotherapy will be effective at increasing serum testosterone levels and improving symptomatology.

## MATERIALS AND METHODS

This was an IRB approved retrospective case series of 44 men (age 26-77) with symptoms of hypogonadism treated by two Andrologists at the University of Miami Miller School of Medicine and the Baylor College of Medicine from February 2015 to May 2018. Of the 44, 9 men were excluded from the study due to concurrent treatment with TRT or Clomiphene Citrate (CC). The men who met inclusion criteria had symptoms of hypogonadism and were ineligible for treatment with TRT with initial T >300 ng/dL, as this implied they did not meet the definition of Testosterone Deficiency. They were prescribed an average of 2000 IU hCG weekly, which is based on the bi/tri-weekly regimen of 1500 IU hCG generally prescribed to men with hypogonadotropic hypogonadism (HH) and infertility, educated on administering it subcutaneously and followed up consistently with proper documentation of follow-up testosterone (T) levels (1). Patients were asked to schedule follow-up clinic visits every 3 months, with labs taken at these times.

On first encounter, patients were evaluated for symptoms associated with low libido, low energy and erectile dysfunction. Patients were asked about sex drive, exercise tolerance, insomnia and difficulty sleeping, and weight gain. Past medical history and patient co-morbidities, as well as management for these were considered. Patients were then sent for lab testing, which included, testosterone (T), luteinizing hormone (LH), follicular stimulating hormone (FSH), estradiol, hematocrit and prostate specific antigen (PSA). Repeat T was taken, and the mean of two samples established baseline and informed diagnosis. The study collected and evaluated all mentioned parameters, but only follow-up T was mandated for inclusion in study. The study also evaluated baseline characteristics such as age, treatment indications, hCG dosage, past medical history and physical exam findings. Dates of each patient’s treatment initiation and the latest follow-up visits were recorded to evaluate the average duration of treatment, and patient reports of side effects, complications and symptom improvement were recorded.

The major parameters of concern for analysis were T improvement from initial to follow-up as well as the degree of T changes as they correlated to dosage and other baseline characteristics such as LH, FSH and therapy duration. The Mann Whitney U test was utilized to compare initial to follow-up T, and a Multiple Linear Regression was used to describe correlation and the significance of correlation between dose, LH, FSH and duration of therapy with percent change in T. All statistical analysis was performed using Microsoft Excel.

## RESULTS

The study included 20 men. Average age was 50.3 (SD 15.6) years and ranged from 26 to 77. On past medical history, 2 of the patients had a history of anabolic steroid use, and two had a history of prostate cancer, one of whom was post-radical prostatectomy. These patients had an average testicular volume of 14.2cc (SD 4.3), and 3 presented with varicoceles, 2 of which were grade II and 1 of which was grade I (Table-1). Indications for treatment were largely attributed to persistent complaints of one or multiple of either low libido, low energy or erectile dysfunction, but also included infertility and insomnia. Patients received an average dose of 2000 IU weekly.

**Table 1:** Summary of baseline characteristics of men included in study.

Sample	n=20
Age in years	50.3 (15.6)
Testes Volume	14.2 (4.3)
Testosterone (ng/dL)	361.8 (158.2)
No Testicular Abnormality	n=17
Varicoceles	n=3
Grade I	n=1
Grade II	n=2
Grade III	n=0
**Past Medical History**	
None	n=6
Hyperlipidemia	n=4
Anabolic Steroid Use	n=2
Prostate Cancer	n=2

Mean and (Standard Deviation)

These men presented with an average initial T of 361.8 ng/dL (SD 158.2), and improved to an average follow-up T of 519.8 ng/dL (SD 265.6). Duration of therapy for these men averaged 6 months, with an average weekly hCG dose of 2000 IU. Over this period, they experienced an average change in T of 60%. One-tail Mann Whitney U test demonstrated this improvement was significant, as the sample of T at baseline was significantly less (p<0.005) than that of follow-up T. This corresponded with 50% of men subjectively reporting symptom improvement. Of the 10 men who reported symptom improvement, only 2 had negative changes in testosterone levels, both by less than 15%.

After performing a multivariable adjusted analysis, the two variables that were statistically associated and positively correlated with percent T changes were hCG dosage (p=0.0005) and duration of therapy (p=0.03). Considering both variables resulted in an R-square value of 0.62, but this still is not a comprehensive explanation of the variance. Patient age and pre-treatment testicular size were not significantly associated with percent T changes.

## DISCUSSION

In our study, we retrospectively evaluated 20 men who were treated for symptoms of hypogonadism with human chorionic gonadotropin (hCG) monotherapy. We presented these patients in terms of their baseline characteristics, and compared pre-treatment and post-treatment Testosterone levels, evaluating the relationship of treatment period testosterone changes with both hCG dosage and duration of therapy. We found that hCG monotherapy, over an average therapy duration of 6 months, significantly improved testosterone levels in this cohort of men. This corresponded with reports of symptom improvement in 50% of patients, with no reports of side effects or complications. We also found a strong relationship between both hCG dosage and duration of therapy with percent testosterone changes. Administration of hCG is effective in improving intratesticular testosterone, while preventing many of the undesirable side effects associated with exogenous testosterone, including testicular atrophy and fertility preservation (4, 10).

HCG’s ability to preserve spermatogenesis, and even improve semen parameters in patients who had been using exogenous testosterone, have been established (10-13). Vicari et al. looked at long term hCG treatment in 17 men with isolated hypogonadotropic hypogonadism, observing a significant increase in testicular volume, in a time-dependent manner, and testosterone, at 15 and 24 months of treatment (13). Similarly, Habous et al. studied a cohort of 282 men with hypogonadism, separating them into 3 arms - CC alone, hCG alone and a combination of hCG and CC. 94 patients from this study received hCG monotherapy with 5000 IU hCG twice weekly and experienced statistically significant increases in T levels at 1 and 3 months (14). However, most of the above-mentioned studies deal with patients who desire fertility, or desire to maintain fertility, as an outcome of treatment with hCG alone.

Our data suggests that hCG can be a safe and efficacious treatment option for patients with symptoms of hypogonadism who do not desire fertility. We observed significant increases in testosterone over an average therapy duration of 6 months. We also observed that this response to treatment was primarily positively correlated with hCG dosage and duration of therapy and lacked association with initial testicular size and patient age. No side effects or complications were reported by the subjects. It should be noted that 6 men who started hCG therapy failed to follow up after the initial 3-month follow-up visit, and therefore fell out of the study.

Further study is needed to investigate the viability of hCG as a monotherapy for symptoms of hypogonadism. Limitations of this study include non-randomized and retrospective design, small sample size, lack of control group, variability in physical exam and dosing regimens between the two Andrologists, variability in hormonal changes between patients, lack of clinically important information to characterize variability in the study population (such as FSH, estradiol and LH) and analysis of follow-up labs with potentially significant implications for safety, such as hematocrit. If repeated, studies can consider use of a standardized patient questionnaire, such as a qADAM questionnaire. The utility of such questionnaires may lie as an adjunct, in establishing a quantifiable baseline clinical presentation that can be followed thereafter. However, it should be recognized that a thorough history and measurement of serum testosterone are equally important in diagnosis and follow-up, due to the lack of specificity of available questionnaires (15). Future studies should observe patients prospectively, use a standardized measure to survey patient improvement and more consistently monitor hematocrit changes in patients on hCG monotherapy, as this could impact treatment safety and patient eligibility. Effect of baseline LH levels on efficacy of therapy may also be important to establish. Of course, although difficult, randomized studies using control groups for comparison are necessary to confirm the clinical and physiological significance of this treatment regimen. Despite these limitations, the current manuscript provides valuable data in proposing the efficacy and safety of hCG monotherapy for men not meeting criteria for testosterone therapy.

## CONCLUSION

Our study indicated that hCG monotherapy is a safe and efficacious treatment option for patients with symptoms of hypogonadism who do not desire fertility or may not have initial testosterone levels greater than 300 ng/dL, significantly improving testosterone levels with no associated reports of complications or side effects. The role of hCG monotherapy in treating these men is promising. Future studies should evaluate changes in hematocrit levels in these patients, as well as the effect that baseline luteinizing hormone may play on response to hCG monotherapy.
